# Patient‐specific CT dosimetry calculation: a feasibility study

**DOI:** 10.1120/jacmp.v12i4.3589

**Published:** 2011-11-15

**Authors:** Thomas Fearon, Huchen Xie, Jason Y. Cheng, Holly Ning, Ying Zhuge, Robert W. Miller

**Affiliations:** ^1^ Department of Diagnostic Imaging and Radiology and the Children's Research Institute Children's National Medical Center Washington, DC; ^2^ George Washington University Medical Center Washington, DC; ^3^ Radiation Oncology Branch, National Cancer Institute, National Institutes of Health Bethesda MD

**Keywords:** CT, radiation dosimetry, treatment planning system, CT organ dose

## Abstract

Current estimation of radiation dose from computed tomography (CT) scans on patients has relied on the measurement of Computed Tomography Dose Index (CTDI) in standard cylindrical phantoms, and calculations based on mathematical representations of “standard man”. Radiation dose to both adult and pediatric patients from a CT scan has been a concern, as noted in recent reports. The purpose of this study was to investigate the feasibility of adapting a radiation treatment planning system (RTPS) to provide patient‐specific CT dosimetry. A radiation treatment planning system was modified to calculate patient‐specific CT dose distributions, which can be represented by dose at specific points within an organ of interest, as well as organ dose‐volumes (after image segmentation) for a GE Light Speed Ultra Plus CT scanner. The RTPS calculation algorithm is based on a semi‐empirical, measured correction‐based algorithm, which has been well established in the radiotherapy community. Digital representations of the physical phantoms (virtual phantom) were acquired with the GE CT scanner in axial mode. Thermoluminescent dosimeter (TLDs) measurements in pediatric anthropomorphic phantoms were utilized to validate the dose at specific points within organs of interest relative to RTPS calculations and Monte Carlo simulations of the same virtual phantoms (digital representation). Congruence of the calculated and measured point doses for the same physical anthropomorphic phantom geometry was used to verify the feasibility of the method. The RTPS algorithm can be extended to calculate the organ dose by calculating a dose distribution point‐by‐point for a designated volume. Electron Gamma Shower (EGSnrc) codes for radiation transport calculations developed by National Research Council of Canada (NRCC) were utilized to perform the Monte Carlo (MC) simulation. In general, the RTPS and MC dose calculations are within 10% of the TLD measurements for the infant and child chest scans. With respect to the dose comparisons for the head, the RTPS dose calculations are slightly higher (10%–20%) than the TLD measurements, while the MC results were within 10% of the TLD measurements. The advantage of the algebraic dose calculation engine of the RTPS is a substantially reduced computation time (minutes vs. days) relative to Monte Carlo calculations, as well as providing patient‐specific dose estimation. It also provides the basis for a more elaborate reporting of dosimetric results, such as patient specific organ dose volumes after image segmentation.

PACS numbers: 87.55.D‐, 87.57.Q‐, 87.53.Bn, 87.55.K‐

## I. INTRODUCTION

Computed tomography (CT) use in the pediatric patient population has witnessed a significant increase^(^ ^1^ ^)^ as diagnostic applications have expanded in concert with tremendous advances in CT technology. A serious concern has been raised over the risk associated with the radiation exposure from these exams in both adult and pediatric patients.^(^ ^1^ ^–^ ^9^ ^)^ The risk is of particular concern in the pediatric patient due to the increased sensitivity,^(^ ^10^ ^)^ as well as the increased opportunity for expressing radiation damage in their lifetime. While patient‐specific organ doses have been recommended by the BEIR VII Committee^(^ ^11^ ^)^ for use in prospective radiation epidemiologic studies of the risk from radiation exposure, very little organ dose information exists for Multi Detector Computed Tomography (MDCT) examinations, especially for the pediatric patient population.

The calculation or estimation of organ dose is quite complex, given the large variation in patient geometry,^(^ ^12^ ^)^ scanner construction, and performance, as well as differences in the technique factors used to generate the images. Methods have been developed to calculate the effective dose,^(^ ^13^ ^–^ ^14^ ^)^ but these methods require knowledge of the dose to radiosensitive organs. Since direct measurement of organ dose *in vivo* is not practical, alternative approaches have been employed. Measurements in anthropomorphic physical phantoms have been conducted utilizing thermoluminescent dosimeters (TLD), as well as solid state detectors (SSD).^(^ ^15^ ^–^ ^19^ ^)^ In general though, physical measurements have the limitation of being point or plane estimates of organ dose, which are not even averaged over the organ of interest. This is an important consideration, particularly when estimating the dose to bone marrow in children, where the red marrow is more evenly distributed. In addition, the variability of patient anatomy may be significant, particularly in the pediatric patient population where rapid growth affects both organ size and position.

Monte Carlo radiation transport simulation has been utilized to estimate organ doses in mathematically stylized phantoms^(^ ^20^ ^–^ ^27^ ^)^ or in cylindrical acrylic phantoms.^(^ ^28^ ^–^ ^29^ ^)^ These simulations are based upon a rigid and very specific patient geometry and bear little relationship to the real world. Tomographic phantoms developed from actual patient data provide a more detailed and realistic representation of the human anatomy. Zankl et al.^(^ ^30^ ^)^ published the first pediatric tomographic computational phantoms derived from CT scans of an infant and a 7‐year‐old child. Organ dose normalized to entrance air kerma for head and chest axial CT scans were calculated.^(^ ^31^ ^)^ Caon et al.^(^ ^32^ ^)^ developed a pediatric phantom called Adelaide after a 14‐year‐old child's CT torso examination. The most recent tomographic phantoms of a 6‐day‐old female and a 2‐month‐old male were developed by Nipper et al.^(^ ^33^ ^)^ and revised by Staton et al.^(^ ^34^ ^)^ Staton et al.^(^ ^35^ ^)^ reported good agreement for Monte Carlo simulations of helical multislice CT (MSCT) scans of the head, chest, abdomen, and pelvis to assess organ and effective dose for the tomographic phantom developed by Nipper et al. and a stylized phantom based upon the Oak Ridge National Laboratory (ORNL) newborn phantom, as revised by Han et al.^(^ ^36^ ^)^ Monte Carlo simulation has been shown to be a very useful tool in the estimation of organ dose in three‐dimensional geometry, since it has the capability to account for density and atomic number variations within the patient by simulating radiation transport and energy deposition events. One of the most widely used Monte Carlo codes for radiation transport calculations is EGSnrc (Electron Gamma Shower).^(^ ^37^ ^)^ This code has been extensively studied.^(^ ^38^ ^–^ ^40^ ^)^ It is impractical, however, to use the code in a routine diagnostic environment. In order to obtain statistically significant results, a sufficiently large number of histories must be simulated requiring substantial computer calculation time.^(^ ^41^ ^–^ ^43^ ^)^

This study was designed to develop and validate a method for estimating organ dose in patients from axial computed tomography based on a radiation treatment planning system (RTPS) that was modified to calculate patient‐specific CT dose distributions. Monte Carlo simulations based on the EGSnrc code were used for modeling radiation transport and detailed machine parameters, such as bowtie filter and cam collimator (provided by GE) for a GE Light Speed Ultra Plus CT scanner. While the characterization of bowtie filtration and beam z‐axis profile using Monte Carlo simulation are not novel ideas,^(^ ^44^ ^,^ ^45^ ^)^ the MC simulations offers an additional independent dose comparison to the RTPS algorithm. The RTPS algorithm is based on a convolution–superposition algorithm utilizing measured beam data along with homogeneity correction applied which, although well established in radiotherapy community, still lacks the rigor provided by MC simulations, as illustrated in the AAPM Report Number 85.^(^ ^46^ ^)^ Experimentally, TLD measurements of dose at specific points (which are representative of organs of interest) in anthropomorphic phantoms were compared with both the RTPS and Monte Carlo calculations of the same virtual phantoms (digitally represented). The advantage of the RTPS approach is the potential for a significant reduction in computation time.

## II. MATERIALS AND METHODS

### A. Radiation treatment planning system (RTPS)

The RTPS has been studied previously for the delivery of external photon and electron beam radiation therapy.^(^ ^46^ ^–^ ^48^ ^)^ Photon dose is calculated utilizing a pencil beam superposition–convolution algorithm which has been well studied in the radiotherapy community.^(^ ^46^ ^)^ The principal concept of the treatment planning algorithm for the determination of the total dose at any point is to consider the contributions of primary and scattered dose components separately.^(^ ^46^ ^,^ ^49^ ^)^ The primary dose component is defined as part of the dose which is deposited by photons that travel directly from the radiation source and are incident upon the surface of the patient, while the scattered dose component is defined as that part of the dose which is deposited by photons which have previously interacted at least once in the medium.^(^ ^46^ ^)^ Numerous algorithms, such as scatter kernels and differential pencil beams, have been developed to determine the scatter dose components.^(^ ^46^ ^)^ In this CT dosimetry study, the algorithm was modified to account for the primary and scatter dose components from kilovoltage level X‐rays generated by CT. The relative dose distribution requires normalization at the CT isocenter (the center of rotation) in order to account for dose contributions from multiple, equally spaced, narrow fan beams entering from a 360° arc with respect to the axial plane. The procedure for dose normalization consists of the determination of the relative dose at the isocenter by integrating the dose distributions of photon beams entering from equal angular increments over the full rotational span. These relative isodose profiles are then normalized by the global maximum dose within the full body scan. The determination of absolute dose at the isocenter of RTPS requires a “calibration factor” that can be determined by a set of standardized measurements under referenced condition, such as the ${\mathrm{CTDI}}_{100}$ at the isocenter. The uncertainty of the RTPS is within 2%.^(^ ^50^ ^)^ The standard deviation for the whole body scan is determined by averaging the standard deviations of multiple X‐ray beams rotated through 360°.

The validation of the RTPS consists of a commissioning process that requires extensive measurements of machine‐specific data, followed by validation with several test cases similar to those performed during the commissioning of a radiotherapy treatment planning system.^(^ ^51^ ^)^ The commissioning of the RTPS requires four sets of measured data specific to each CT scanner. These include normalized depth dose curves and crossbeam profiles in both the trans‐axial (along the x axis) and sagittal (along the z‐axis) plane, as well as the calibration to absolute dose at the normalization point (CT isocenter). These measurements depend upon X‐ray energy (kVp), beam width, and type of bowtie filter.

**Figure 1:**
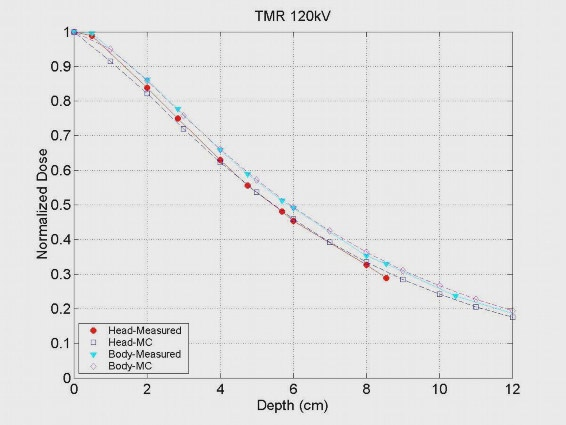
TMR of GE Light Speed Ultra Plus CT scanner for measurement and Monte Carlo simulation.

In this study, a GE Light Speed Ultra Plus CT scanner (GE Healthcare, Waukesha, WI) was used, with a potential of 120 kVp, two types of “bowtie filters” (head and body), and beam width of 5, 10, 15, and 20 mm. The first required data represent the variation of dose with depth. These can be measured as either percentage depth doses (PDD) or tissue maximum ratios (TMR).^(^ ^52^ ^)^ Depth dose curves at 120 kVp and the head and body “bowtie filters” were measured using a parallel plate ionization chamber (PTW Freiburg N23342‐1123) in a solid water phantom. The solid water phantom (Model 457 Gammex RMI, Middleton, WI) has a dimension of 30 by 30 cm with slab thicknesses varying from 0.2 to 6 cm. The dosimetric properties and transmission values of a solid water phantom have been well characterized by both measurement and MC simulation.^(^ ^53^ ^,^ ^54^ ^)^ Although many facilities may use reduced kVp setting for pediatric patients, the 120 kVp was selected as a proof of concept, and this energy setting was constant throughout the measurements and calculations. Figure 1 illustrates the TMR for measurements at 120 kVp, for body and head “bowtie filters”, using a 10 mm beam width setting.

The next set of required data are crossbeam profiles measured at the surface of the solid water phantom in both the trans‐axial (along the x‐axis) and sagittal (along the z‐axis) plane, in the presence of both “bowtie filters” at four beam width settings (5, 10, 15, and 20 mm). These measurements were performed using film dosimetry (Kodak Diagnostic Film Ready‐Pack X‐Omat TL 2 cat no. 810–1537) and a VIDAR VXR‐16 densitometer. For the trans‐axial and sagittal beam profiles, the Ready‐Pack film was positioned at isocenter on 7.62 cm (3″) of Styrofoam. The X‐ray tube was oriented vertically downward and a single exposure was taken for each beam width (5, 10, 15, and 20 mm). The films were scanned with a VIDAR VXR‐16 densitometer calibrated to the film's exposure response curve. The optical density was converted to exposure and the beam profiles were normalized to the maximum response.

The sagittal profile measurement was also verified by means of ion chamber measurements using a CT pencil chamber (10x5‐3CT pencil chamber, Radcal Corp., Monrovia, CA). The chamber was positioned at isocenter with the X‐ray tube pointing vertically down. The chamber was manually displaced along the x‐axis in 1 cm increments with an exposure taken at each location and normalized with respect to the central reading (isocenter). Measurements were made for both the head (SFOV=25 cm) and the body (SFOV=50 cm) bowtie filters.

**Figure 2:**
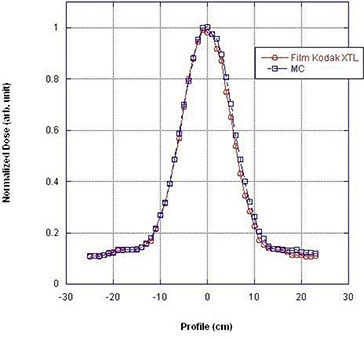
Transaxial (x‐axis) beam profile: 120 kV, head bowtie filter, and 10 mm beam width.

**Figure 3:**
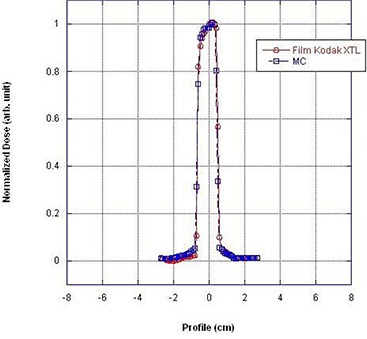
Longitudinal (z‐axis) beam profile: 120 kV, head bowtie filter, and 10 mm beam width.

The crossbeam profiles were normalized at the center of the profile (as defined by the Full Width at Half Maximum). Figures 2 and 3 illustrate the crossbeam dose profiles measured in a single static shot at 120 kVp, head “bowtie filter” and 10 mm beam width in the trans‐axial and sagittal plane, respectively. The “heel effect” of the dose profile is illustrated in Fig. 3 and is consistent with results reported by Imaging Performance Assessment of CT Scanners (ImPACT), London, UK.^(^ ^55^ ^)^

**Figure 4:**
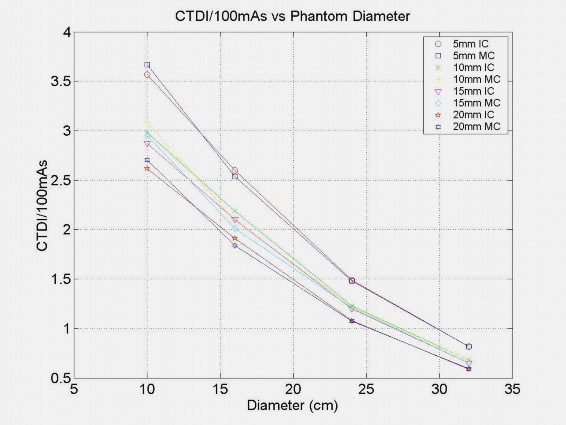
Reference ${\mathrm{CTDI}}_{100}$ center dose measurement with ion chamber (IC) and Monte Carlo (MC) calculation as a function of cylindrical phantom diameter.

The last required dataset was reference dose measurements used for absolute dose calibration. The methodology used for determination of the conversion factor from normalized measurements to absolute dose was performed by measurement of ${\mathrm{CTDI}}_{100}$,^(^ ^56^ ^)^ whereby a cylindrical ionization chamber (Radcal model 10x5‐3CT) is placed at the center of cylindrical PMMA phantoms of diameters of 10 cm, 16 cm, 24 cm, and 32 cm. The sensitive volume of the ionization chamber was $3\;{\text{cm}}^{3}$, with trans‐axial length of 10 cm. The ionization chamber measurements were performed under a number of machine‐specific beam widths (5, 10, 15, and 20 mm). These measurements provide a reference table of dose as a function of depth relative to CT isocenter. Intervening data points were interpolated from the measurements. If the selected isocenter is not exactly at the center of the object of interest, or if the contour of the object of interest is not circular, then an average depth of isocenter was used. This depth was determined by averaging the distances between isocenter and the surface over a full 360° rotation with respect to the axial plane. Figure 4 illustrates the reference dose for the cylindrical phantoms as a function of phantom diameter in the CTDI measurements. Beam modeling of the CT dosimetry system was performed upon completion of the machine data measurements. It should be noted that, although the CT dosimetry can be simulated with any number of photon beams, the isodose profiles showed no significant difference (<1%) between simulations of 16 and 32 beams entering the virtual phantom from 360° in equal angular increments.

### B. Monte Carlo simulation

Monte Carlo (MC) simulations were performed using EGSnrc codes to examine CT radiation dose distributions. The codes utilize BEAMnrc for simulation of the X‐ray generation with electrons on target, DOSXYZnrc for calculation of virtual phantom dosimetry, CTCREATE for conversion of CT image data, as well as EGSnrc for the MC calculation kernel.

BEAMnrc was used to simulate the GE CT scanner parameters (GE Light Speed Ultra Plus) including geometries and composition of the X‐ray tube, energy spectral distribution, “bowtie” filters, and z‐axis collimation (obtained under a nondisclosure agreement between General Electric Medical Systems and the authors). The simulation parameters for the photon transport cutoff energy (PCUT) and for the electron transport cutoff energy (ECUT) were 1 keV and 10 keV, respectively. For improved accuracy of low‐energy simulation, the spin effect, Koch‐Motz bremsstrahlung angular sampling, National Institute of Standards and Technology (NIST) cross‐section data, Sauter photoelectron angular sampling, Rayleigh scattering and atomic relaxation were all applied to BEAMnrc parameters.^(^ ^37^ ^,^ ^57^ ^)^ To enhance simulation efficiency, variance reduction techniques of directional bremsstrahlung splitting (DBS) and Russian roulette were applied.^(^ ^37^ ^)^ A splitting number of 2000 was used for DBS. Phase‐space files containing lists of particle history including position, energy, direction, charge, weight, and last interaction of each particle were stored. These phase‐space files were then used for the determination of dose to a region‐of‐interest.

DOSXYZnrc was used to determine the relative dose deposited per simulated particle (MeV/ particle) in cylindrical and anthropomorphic virtual phantoms, as well as patient‐specific CT scans. The X‐ray beam was rotated through 360°. The relative dose was normalized by global maximum within the full body scan. The dose calibration was performed similarly to the RTPS by converting normalized values to absolute dose at isocenter. The standard deviation of each X‐ray beam was based on the history‐by‐history method, which depends upon the number of simulated particles (number of independent events).^(^ ^37^ ^,^ ^58^ ^)^ The standard deviation for the whole body scan was determined by averaging the standard deviation of multiple X‐ray beams rotated through 360°. CTCREATE was used to convert CT images in DICOM format into a CT‐based virtual phantom using a voxel size of 1 by 1 by $4\;{\text{mm}}^{3}$. A CT electron density phantom (Model RMI‐465 Gammex RMI, Middleton, WI) was used to generate a conversion curve of Hounsfield numbers to electron densities. The Monte Carlo simulations were performed on a computer cluster composed of six computers each with a single core (Dell Precision 340, Pentium I V, 1.7GHz), with an average simulation time of approximately 96 hours for each 16 beam segmented slice.

### C. Thermoluminescent dosimeter (TLD) measurements

Dose measurements were performed in two pediatric anthropomorphic phantoms (Humanoid Systems, Carson, CA) using thermoluminescent dosimeters (TLDs) for the verification of the dose calculation generated by the CT dosimetry system. These anthropomorphic phantoms have anatomic features of the chest and skull of both a newborn and a 12‐year‐old child. ${\mathrm{CaF}}_{2}$:Mn (TLD‐200, Harshaw, Thermo Fisher Scientific Inc., Waltham, MA) thermoluminescent chips (dimensions of 3.2 by 3.2 by $0.89\;{\text{mm}}^{3}$) were used for the dose measurements. Five TLD chips were placed in holes for each (anatomically represented) organ structure of each phantom. Digital representations of the physical phantoms (virtual phantoms) were then generated from CT scans under the following scan parameters: 120kVp, 100mA, 1 second rotational time, 10 mm beam width, axial mode and large or small SFOV. The chest/abdomen images were scanned between the clavicle and the sacrum. The head images were scanned between the top and the base of the skull. The luminescent signals were measured by an automated TLD reader (Model 5500, Harshaw), and the average of five readings is reported and compared with dose values obtained from RTPS and MC calculations. The dose to the skull is an average of four measurement locations (AP, PA, and right/left lateral). The dose calculations from RTPS and MC are determined from calculations of the virtual anthropomorphic phantoms with the TLDs present. The location of the TLDs in the image set characterizes discrete voxel volumes. The isodose values at these positions were averaged to determine the calculated dose values for the RTPS and MC.

## III. RESULTS

Figure 1 demonstrates tissue maximum ratios (TMR) as a function of depth in a solid water phantom (measured) and a virtual phantom (calculated by Monte Carlo). Figures 2 and 3 illustrate the excellent correlation of measured and simulated (MC) trans‐axial and longitudinal beam profiles for the head “bowtie filter” at the CT isocenter. Figure 4 presents measured and calculated (MC) values of the ${\text{CTDI}}_{100}/100$ mAs as a function of the diameters of cylindrical phantoms positioned at the center of CT rotation. This figure indicates that the dose to pediatric patients relative to adults will increase for a given mAs — which reinforces the need to adjust technique factors specific to the patient.

**Figure 5:**
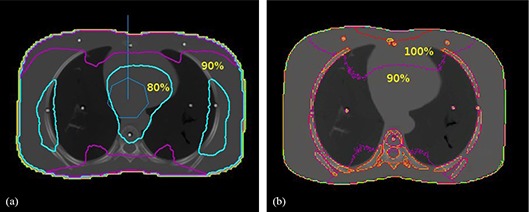
Transaxial view of a chest image of a child phantom with isodose lines superimposed: (a) and (b) illustrate the isodose curves calculated by RTPS and MC, respectively. The solid bright dots represent the locations of the TLD measurements. The hexagonal structure with a vertical line represents the location of the isocenter of the circular CT fan beam.

**Figure 6:**
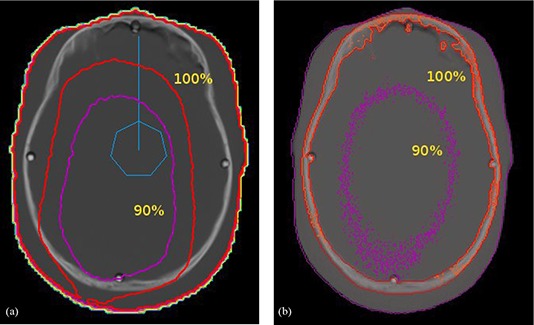
Transaxial views of a head image of a child phantom with isodose lines superimposed: (a) and (b) illustrate the isodose curves calculated by RTPS and MC, respectively. The solid bright dots represent the locations of the TLD measurements. The hexagonal structure with a vertical line represents the location of the isocenter of the circular CT fan beam.

TLD dose measurements were performed in anthropomorphic phantoms for the validation of the RTPS dose calculation algorithm. Tables 1 and 2 illustrate the absorbed dose to the chest of a newborn and a 12‐year‐old child, respectively, expressed in units of mGy/100mAs, comparing TLD measurements in anthropomorphic phantoms and calculations in anthropomorphic virtual phantoms by RTPS and Monte Carlo. Similar data for the skulls of a newborn and a 12‐year‐old child are presented in Tables 3 and 4. These measurements were taken using a whole body scan, with a scan technique of 120 kVp, head and body “bowtie filters”, in axial mode, and a beam width of 10 mm. For illustrative purpose, Fig. 5 shows a trans‐axial view of the virtual chest phantom overlaid with isodose curves from the RTPS and MC calculations with the solid bright spots representing the TLD positions. Similarly, Fig. 6 shows trans‐axial views of a head image of a child phantom with isodose lines superimposed from RTPS and MC calculations. It is worth noting that the isodose distribution in Fig. 6(b) is not symmetric, due to the position of the isocenter within the phantom. The hexagonal structure with a vertical line represents the location of the isocenter of the circular CT fan beam. The RTPS calculation utilizes the isocenter of the CT scan to calculate the dose. As illustrate in Fig. 6(b), the isocenter is closer to one side of the head and, as a result, is “hotter” (as indicated by the red isodose curves). This result indicates that there are important positioning implications for abdominal scans of pregnant patients, and suggests that the technologist should move the isocenter away from the pelvic region in order to minimize the dose to the unborn fetus.

**Table 1:** Dose (mGy/100mAs) in newborn chest phantom for TLD measurements, treatment planning system calculations (RTPS), and Monte Carlo (MC) calculations. The percentage dose differences (normalized to TLD measurements) between the TLD measurements and the RTPS and MC calculations are shown in the third, fifth and the sixth columns, respectively. The percentage dose differences (normalized to MC measurements) between the RTPS and MC calculations are shown in the last two columns.

	*Dose TLD*	*Dose RTPS*	*% Diff. TLD/RTPS*	*Dose MC*	*% Diff. MC/TLD*	*% Diff. MC/RTPS*
Left Lung	30.7 (±2.0)	29.2 (±0.3)	4.9	30.8 (±0.5)	0.3	5.2
Right Lung	28.8 (±2.3)	27.6 (±0.3)	4.2	29.3 (±0.5)	1.7	5.8
Left Rib	31.4 (±2.4)	31.5 (±0.3)	0.3	32.5 (±0.5)	3.5	3.1
Right Rib	27.3 (±1.5)	29.2 (±0.3)	7.0	31.1 (±0.5)	13.9	6.1
Sternum	32.8 (±2.1)	31.1 (±0.3)	5.2	30.8 (±0.5)	6.1	1.0
Left Breast	28.9 (±2.6)	32.3 (±0.3)	11.8	31.1 (±0.5)	7.6	3.9
Right Breast	30.8 (±3.0)	32.7 (±0.3)	6.2	31.5 (±0.5)	2.3	3.8

**Table 2:** Dose (mGy/100mAs) in child chest phantom for TLD measurements, treatment planning system calculations (RTPS), and Monte Carlo (MC) calculations. The percentage dose differences (normalized to TLD measurements) between the TLD measurements and the RTPS and MC calculations are shown in the third, fifth, and sixth columns, respectively. The percentage dose differences (normalized to MC measurements) between the RTPS and MC calculations are shown in the last column.

	*Dose TLD*	*Dose RTPS*	*% Diff. TLD/RTPS*	*Dose MC*	*% Diff. MC/TLD*	*% Diff. MC/RTPS*
Left Lung	20.2 (±1.2)	18.9 (±0.1)	6.4	19.7 (±0.8)	2.5	4.1
Right Lung	21.8 (±1.2)	19.9 (±0.1)	8.7	20.4 (±0.8)	6.4	2.5
Left Rib	19.7 (±1.0)	22.0 (±0.1)	11.7	18.5 (±0.8)	6.1	18.9
Right Rib	20.7 (±1.3)	22.8 (±0.1)	10.1	19.2 (±0.8)	7.2	18.8
Sternum	25.9 (±1.6)	26.4 (±0.2)	1.9	22.0 (±0.8)	15.1	20.0
Left Breast	22.8 (±1.3)	20.6 (±0.5)	9.6	19.0 (±0.8)	16.7	8.4
Right Breast	23.8 (±1.4)	20.8 (±0.4)	12.6	19.2 (±0.8)	19.3	8.3

**Table 3:** Dose (mGy/100mAs) in newborn skull phantom for TLD measurements, treatment planning system calculations (RTPS), and Monte Carlo (MC) calculations. The percentage dose differences (normalized to TLD measurements) between the TLD measurements and the RTPS and MC calculations are shown in the third, fifth, and sixth columns, respectively. The percentage dose differences (normalized to MC measurements) between the RTPS and MC calculations are shown in the last column.

	*Dose TLD*	*Dose RTPS*	*% Diff. TLD/RTPS*	*Dose MC*	*% Diff. MC/TLD*	*% Diff. MC/RTPS*
Skull	23.8 (±1.7)	28.1 (±0.3)	18.1	25.3 (±0.4)	6.3	11.1
Left Eye	24.7 (±1.4)	30.3 (±0.3)	22.7	24.0 (±0.4)	2.8	26.3
Right Eye	24.1 (±1.5)	30.0 (±0.3)	24.5	23.9 (±0.4)	0.8	25.5
Jaw	26.8 (±1.9)	29.1 (±0.3)	8.6	29.1(±0.4)	8.6	0.0

**Table 4:** Dose (mGy/100mAs) in child skull phantom for TLD measurements, treatment planning system calculations (RTPS), and Monte Carlo (MC) calculations. The percentage dose differences (normalized to TLD measurements) between the TLD measurements and the RTPS and MC calculations are shown in the third, fifth, and sixth columns, respectively. The percentage dose differences (normalized to MC measurements) between the RTPS and MC calculations are shown in the last column.

	*Dose TLD*	*Dose RTPS*	*% Diff. TLD/RTPS*	*Dose MC*	*% Diff. MC/TLD*	*% Diff. MC/RTPS*
Skull	18.9 (±1.1)	22.8 (±0.2)	20.6	23.1 (±0.6)	22.2	1.3
Left Eye	22.3 (±1.3)	24.2 (±0.2)	8.5	23.7 (±0.6)	6.3	2.1
Right Eye	20.9 (±1.3)	22.7 (±0.2)	8.6	22.8 (±0.6)	9.1	0.4
Jaw	18.9 (±1.0)	22.4 (±0.2)	18.5	21.8 (±0.6)	15.3	2.8

## IV. DISCUSSION

The main goal of this study was to provide a proof of concept for implementing a patient‐specific CT dosimetry system utilizing the methodologies employed for radiotherapy treatment planning software. In general, the RTPS and MC dose calculations are within 10% of the TLD measurements for both the infant and child chest scans. With respect to the dose comparisons for the head, the RTPS dose calculations are slightly higher (10%–20%) than the TLD measurements, while the MC results are within 10% of the TLD measurements. One of the factors that can contribute to the discrepancy between these two sets of data is the environment in which the TLD measurements are made. In the anthropomorphic phantoms, there is a small air gap between each of the slabs of the phantom, which could not be completely eliminated. Hence in the CT images, there are visible streak marks between the slices of the virtual phantoms. Upon conversion of the CT numbers to electron density, these gaps could lead to an inaccurate dose calculation from the RTPS. In addition, the air gap observed between physical phantom slabs could also result in increased exposure to the TLDs due to the decreased amount of attenuation present. Centering the TLDs in each slab and filling the remaining volume with tissue‐equivalent material helped minimize this effect. We are still investigating the discrepancy between the RTPS and MC results. The RTPS algorithm is based on measured data (depth dose and cross profiles), while MC algorithm is based on probability distributions of individual particle interactions. Perhaps the RTPS dose absorption algorithm needs additional correction.

Although there are no direct comparisons, a number of studies have reported CT dosimetry for varying types of pediatric phantoms (stylized or tomographic) and CT scanners. Nishizawa et al.^(^ ^59^ ^)^ also reported TLD (BeO) measurements of a pediatric phantom (6 year old) using a GE Light Speed scanner operating at 120 kVp. In their study, the tube current was varied from 50 to 200 mA, the exposure time from 0.5 to 0.8 sec, the pitch factor from 0.765 to 1.375, and the beam dimension 5 by $4\;{\text{mm}}^{2}$. This resulted in organ‐specific dose ranges from 16.6 to 21.9 mGy for the lung and 14.2 to 19.8 mGy for the breast. These numbers are consistent with the TLD measurements in this study, taking into account the differences in the phantom, manufacturer, and scanner settings. Similar studies have also reported TLD measurements in cylindrical acrylic phantoms representative of pediatric patients.^(^ ^29^ ^,^ ^60^ ^)^

Although voxel‐based Monte Carlo computation of CT phantoms potentially provides a more accurate method of dose determination, the normalization of units between measurement and calculation can result in significant differences in the dose estimation. Caon et al.^(^ ^61^ ^)^ reported on a CT dosimetry study for a 14‐year‐old female tomographic phantom. The EGS4 radiation transport code was used for Monte Carlo simulation of a GE Hi‐Speed Advantage CT scanner operating at 120 kVp with 10 mm beam width. By using voxel representation, each specific organ can be segmented to allow for a more accurate dose estimation. The Caon study determined the average lung and breast doses to be 5.74 and 5.44 mGy/100mAs, respectively. The authors noted, however, that the lower estimated dose values compared with other studies^(^ ^62^ ^,^ ^63^ ^)^ may be due to the greater than 30% difference in the CTDI value which is used as a normalization factor between measurement and calculation. A number of other studies^(^ ^27^ ^,^ ^31^ ^,^ ^64^ ^)^ have also reported Monte Carlo CT dosimetry on pediatric phantoms. However, the results of these studies are reported in terms of normalized effective doses, thus making them difficult to compare with the current study.

More recently, studies employing the University of Florida (UF) phantoms have been reported by Stanton et al.^(^ ^35^ ^)^ and Lee et al.^(^ ^65^ ^)^ The UF phantom set, which consists of a newborn, a 9‐month‐old male, a 4‐year‐old female, an 8‐year‐old female, an 11‐year‐old male, and a 14‐year‐old male, are tomographically‐based. These phantoms provide a better anatomical representation than do the ORNL phantoms. Staton et al. used the EGSnrc radiation transport code to simulate a Siemens SOMATOM Sensation 16 CT (MSCT) scanner. The Monte Carlo calculations were performed at 120 kVp, using a 10 mm beam width, in axial mode, with a collimator setting of 16 by $0.75\;{\text{mm}}^{2}$ and were normalized to ${\mathrm{CTDI}}_{100}$ measurements. The calculated lung dose for the newborn phantom was determined to be 15.48 mGy/100mAs. Lee et al. used the Monte Carlo N‐Particles (MCNPX) transport code to simulate the same Siemens MSCT scanner under the same CT conditions and also normalized to ${\mathrm{CTDI}}_{100}$ measurements. They reported lung doses of 14.97, 12.04, and 12.59 mGy/100mAs, respectively, for the 9‐month‐old, 11‐year‐old, 14‐year‐old male phantoms. For a 4‐year‐old female phantom, lung and breast doses of 13.85 and 10.08 mGy/100mAs were reported. The estimated doses for lung and breast are slightly lower than the values reported from this study. This difference can be attributed to the volume of the body being scanned. In this study, the doses for each organ were determined from a whole body scan, while the UF phantom studies only employed a region‐of‐interest (head, chest, abdomen, and pelvis) in determining organ dose. Consequently, the scattered dose from other parts of the body contributes to the higher dose value in the current study.

Patient‐specific organ dose determination, as recommended by the BEIR VII Committee, is starting to be reported. Li et al.^(^ ^66^ ^)^ reported a Monte Carlo CT dosimetry study of seven pediatric patients ranging in age from one to six years old. The technique factors for their GE LightSpeed VCT scanner were 120 kVp, 70 or 75 mA, and 0.4 s, with a pitch of 1.375, and 20 mm beam collimation. By segmenting the organs, they reported dose values ranging from 10.4–12.6 for the lung and 7.2–11.7 mGy/100mAs for the breast. Although patient‐specific Monte Carlo CT dosimetry yields the most accurate calculation, the amount of time needed for computation is impractical in a clinical setting. The principal advantage of the RTPS system is the speed of the dose calculation algorithm, typically taking only a few minutes to generate a full dose volume.

## V. CONCLUSIONS

This paper reports a patient‐specific CT dosimetry program based on a modified radiation treatment planning system. The dose calculations using this program have demonstrated results consistent with both TLD measurements and Monte Carlo calculations for both newborn and 12‐year‐old anthropomorphic phantoms. The advantage of the RTPS is the significant reduction in computation time, yielding dose estimates within 10%–20% of measured values. Future research will focus on improving the agreement between measurement and calculation, as well as the implementation of RTPS calculations for helical CT scans.

## ACKNOWLEDGMENTS

The authors would like to thank GE Medical Systems for providing detailed information of the GE LightSpeed CT scanner. This research was supported by the Intramural Research Program of the National Cancer Institute, NIH.
